# Mutation of Aspartate 238 in FAD Synthase Isoform 6 Increases the Specific Activity by Weakening the FAD Binding

**DOI:** 10.3390/ijms20246203

**Published:** 2019-12-09

**Authors:** Piero Leone, Michele Galluccio, Stefano Quarta, Ernesto Anoz-Carbonell, Milagros Medina, Cesare Indiveri, Maria Barile

**Affiliations:** 1 Department of Biosciences, Biotechnology and Biopharmaceutics, University of Bari, via Orabona 4–, 70126 Bari, Italy; pieroleone87@gmail.com (P.L.); quartastefano93@gmail.com (S.Q.); 2 Department of Biology, Ecology and Earth Sciences (DiBEST), Unit of Biochemistry and Molecular Biotechnology, University of Calabria, via P. Bucci 4c, 87036 Arcavacata di Rende, Italy; michele.galluccio@unical.it (M.G.); cesare.indiveri@unical.it (C.I.); 3 Departamento de Bioquímica y Biología Molecular y Celular, Facultad de Ciencias, Instituto de Biocomputación y Física de Sistemas Complejos (GBsC-CSIC and BIFI-IQFR Joint Units), Universidad de Zaragoza, 50009 Zaragoza, Spain; eanoz@unizar.es (E.A.-C.); mmedina@unizar.es (M.M.)

**Keywords:** FAD synthase, FMN adenylyl transferase, FADS isoform 6, supermutant

## Abstract

FAD synthase (FADS, or FMN:ATP adenylyl transferase) coded by the FLAD1 gene is the last enzyme in the pathway of FAD synthesis. The mitochondrial isoform 1 and the cytosolic isoform 2 are characterized by the following two domains: the C-terminal PAPS domain (FADSy) performing FAD synthesis and pyrophosphorolysis; the N-terminal molybdopterin-binding domain (FADHy) performing a Co^++^/K^+^-dependent FAD hydrolysis. Mutations in FLAD1 gene are responsible for riboflavin responsive and non-responsive multiple acyl-CoA dehydrogenases and combined respiratory chain deficiency. In patients harboring frameshift mutations, a shorter isoform (hFADS6) containing the sole FADSy domain is produced representing an emergency protein. With the aim to ameliorate its function we planned to obtain an engineered more efficient hFADS6. Thus, the D238A mutant, resembling the D181A FMNAT “supermutant” of *C. glabrata*, was overproduced and purified. Kinetic analysis of this enzyme highlighted a general increase of K_m_, while the k_cat_ was two-fold higher than that of WT. The data suggest that the FAD synthesis rate can be increased. Additional modifications could be performed to further improve the synthesis of FAD. These results correlate with previous data produced in our laboratory, and point towards the following proposals (i) FAD release is the rate limiting step of the catalytic cycle and (ii) ATP and FMN binding sites are synergistically connected.

## 1. Introduction

The riboflavin (Rf) derived FMN and FAD cofactors play a pivotal role in cell economy ensuring the functionality of the flavoproteome, mainly localized in mitochondria [1,2]. Consistent with the crucial role of flavins and flavoenzymes in cell life, several diseases, including neuromuscular and neurological disorders are linked to flavin-dependent enzyme deficiency or impairment in Rf homeostasis in humans and experimental animals. These disorders, in some cases, can be cured with high doses of Rf, as the two Rf-responsive (RR) disorders Brown–Vialetto van Laere syndrome (BVVLS) [3,4] and RR-multiple acyl-CoA dehydrogenase deficiency (RR-MADD) [5,6,7]. In mammalian cells, Rf is taken up via translocators (SLC52A1-3, also named RFVT1-3) [8,9] and converted into FMN and FAD via the sequential action of Rf kinase (RFK, EC 2.7.1.26) and FAD synthase or FMN-ATP adenylyl transferase (FADS, EC 2.7.7.2).

The only gene identified for coding functional FAD synthases in humans is FLAD1 gene (GenBank, A. N. DQ458779, [10]) localized on chromosome 1, which is orthologue of *flad-1* in *C. elegans*, [11], Fad1 in *S. cerevisiae* [12] and *FMNAT* in *C. glabrata* [13]. The structures of FADSs from yeast, but not that of the human orthologue, have been solved [13,14].

The FLAD1 gene in humans generates different alternative transcript variants, with unknown differential expression profile, producing protein isoforms, only in part characterized, which have different subcellular localization [15] and domain organizations.

The most abundant variant in all the tissues and cells tested so far, is isoform 2 (NM_201398.3 in NCBI GenBank), which corresponds to a cytosolic enzyme of 490 amino acids [15]. The FADS or FMN-AT module of this protein is localized in the C-terminus of the protein; it contains a phosphoadenosine 5-phosphosulfate (PAPS) reductase domain and it is fused with a molybdopterin binding resembling (MPTb) domain located in the N-terminus [16,17].

FAD synthesis catalyzed by hFADS2 follows a bi-bi ordered kinetics with ATP entering prior to FMN and pyrophosphate released before FAD [17]. This enzyme contains 10 cysteines, some of which are relevant for catalysis; two of them are stably reduced (C139 and C241, one for each protein domain), four are implicated in stable disulfide bridges (C399 to C402, C303 to C312, both in the PAPS domain), and the other four are forming redox sensitive disulfides (C39 to C50; C440 to C464) [18].

Following the discovery that hFADS2 is a bifunctional enzyme, with the N-terminal domain working as a Co^2+^-dependent FAD hydrolase, the two domains of the protein were renamed as FADHy and FADSy, functionally corresponding to E.C. 3.6.1.18 and E.C. 2.7.7.2, respectively [19,20]. FADHy is also present in the other three isoforms of the protein reported in the NCBI GenBank (FLAD1 isoform 1, NM_025207.5; FLAD1 isoform 3, NM_001184891.2; and FLAD1 isoform 4, NM_001184892.2). Nonetheless, it is indeed absent in the FAD1p yeast counterparts and, more interestingly, in another human isoform not yet annotated as FADS, but reported as CRA_d in NCBI and known as hFADS6.

The isoform hFADS6 is a 320-residue long protein, containing the sole FADSy domain, whose corresponding transcript was recently described in the frame of studying FLAD1 mutations leading to RR-MADD [7]. The relevance of this novel isoform lies in its ability to ensure FAD supply to patients carrying frameshift mutations in exon 2 of the FLAD1 gene and, for this reason, it has been named an “emergency protein” for MADD patients. When produced in *E. coli* and purified at homogeneity, hFADS6 behaves as a yellow monomer, able to tightly, but not covalently, bind FAD. Recombinant hFADS6 is more stable than hFADS2 and is able to perform FAD synthesis starting from FMN and ATP. As expected, it is not able to perform FAD hydrolysis [21]. The molecular features of this novel natural form well correlate with those of a previously produced artificial construct, lacking the first 231 residues of hFADS2, which per se can fold and catalyze the FAD synthesis reaction [22]. Therefore, this novel isoform of FAD forming enzymes is, in our opinion, a good model to address remaining challenges in the catalytic behavior of the FAD synthesis reaction in humans, as compared with the our deeper understanding of the yeast orthologues [13,14], from which structure the human protein was modeled [21]. The more striking point concerning the catalytic cycle of FAD forming enzyme concerns the observation that the turn-over number of the reaction, as catalyzed by hFADS2, is quite low (0.069 ± 0.011 s^−1^), with FAD release being the limiting step of the over-all reaction [23]. This apparently sounds strange for a protein which is expected to be devoted to FAD delivery. We postulate that redox events or protein–protein interaction in a sort of chaperoning process may promote cofactor delivery to cognate apo-flavoprotein [17,23].

The aim of this work was to confirm the proposed mechanism by proving that lowering the FAD affinity toward the catalytic site (i.e., facilitating FAD release) results in increasing the turn-over number of the FAD forming reaction. To obtain these results we took into account studies on *Candida glabrata* FMN-AT (*Cg*FMN-AT) [24] that identified a cryptic residue, precisely D181, whose mutation to Ala resulted in an increase of the V_max_, and therefore it was named “super-mutant”. The orthologous residue in the human enzyme is D238. We investigate, here, the effects of the D238A mutation on hFADS6 steady-state activity and binding capability to confirm our hypothesis that a weak FAD binding to the active site could led to an increased rate of synthesis. The possible final goal of this study is to open a perspective towards increasing the emergency enzyme activity in patients suffering for *FLAD1* mutations.

## 2. Results

### 2.1. Homology Model of D238A-hFADS6

**Figure 1:**
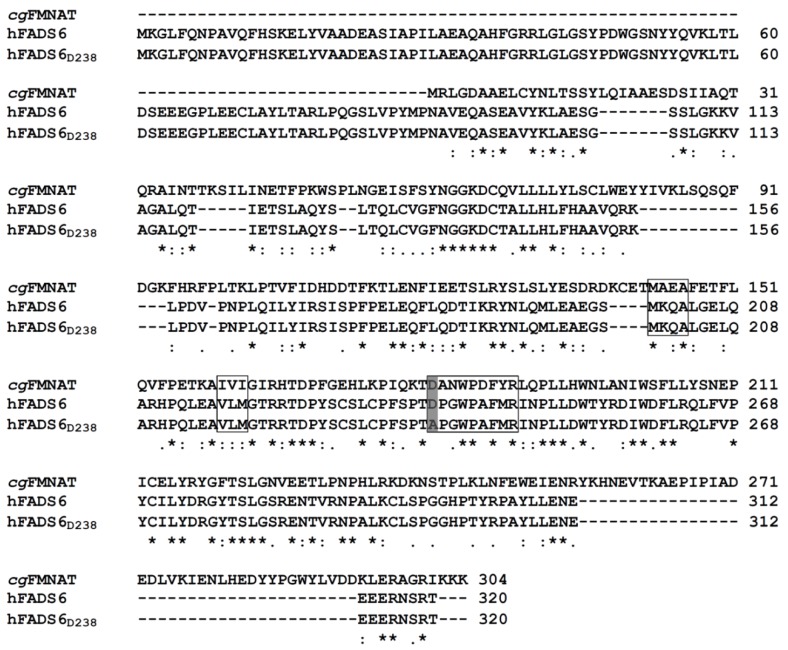
Sequence alignment of *Cg*FMN-AT (Q6FNA9), hFADS6, and hFADS6_D238A_. The protein sequences were aligned by Clustal Omega software. D181 of the yeast protein, D238 of the hFADS6, and A238 in the mutant are shadowed in grey. Amino acids forming the flavin binding motif are highlighted by boxes.

The amino acid sequences of WT and D238A mutant hFADS6 were aligned by Clustal Omega software with the FMN-AT protein Q6FNA9 of *C. glabrata*. The alignment presented in Figure 1 highlights not only a high percentage identity (about 32%) between the human and the yeast proteins, but also confirms that D238 of hFADS6 corresponds to D181 of the yeast orthologue and that it is part of the flavin binding motif.

**Figure 2:**
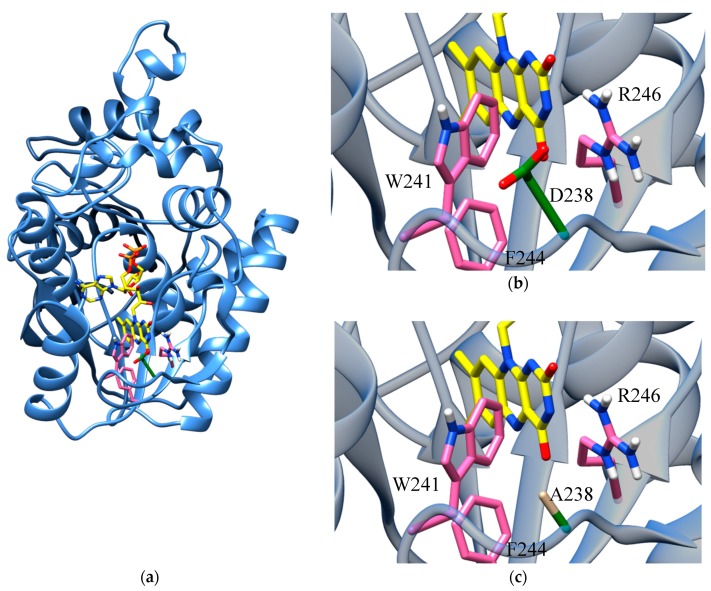
Modeling and FAD docking of hFADS6: (**a**) Ribbon representation of hFADS6 modeled as described in [21]. FAD transferred in the active site from PDB ID: 3G6K and represented as yellow licorice colored by heteroatom type. (**b**,**c**) Zoom into the binding site of the isoalloxazine moiety of FAD. Amino acids involved in isoalloxazine binding are colored in pink. D238 in the WT (**b**) and A238 in the mutant (**c**) are rendered as green licorice colored by heteroatom type.

Due to the lack of suitable templates for modeling the N-terminus (amino acids 1 to 108) of hFADS6, an ab initio strategy was previously adopted to obtain its three-dimensional (3D) structural model [21]. FAD was then inserted in the active site according to the structure of *Cg*FMN-AT (3G6K) as described in Materials and Methods. Figure 2 clearly shows a reduced steric hindrance upon substitution of D238 with A. However, the major reason for varied FAD binding and release kinetics should be the loss of the dipole interaction between the Asp-carboxylate group and the N(3)H-FAD, which most affects FAD binding.

### 2.2. Cloning, Expression, and Purification of the D238A-hFADS6 Isoform

**Figure 3:**
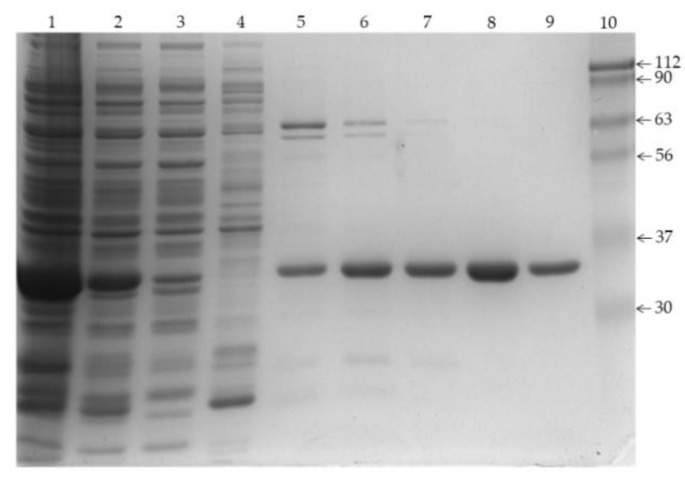
Protein fractions obtained by Ni^2+^-chelating chromatography were separated by SDS–PAGE on 12% polyacrylamide gel and stained with Coomassie blue. Lane 1, insoluble fraction of IPTG-induced cell lysate (6 μg); lane 2, soluble fraction of IPTG-induced cell lysate (28 μg); lane 3, first flow-through fraction (19 µg), lane 4, proteins eluted with 50 mM imidazole (13 µg), lane 5, first fraction of proteins eluted with 100 mM imidazole (2 µg), lane 6, second fraction of proteins eluted with 100 mM imidazole (3 µg), lane 7, third fraction of proteins eluted with 100 mM imidazole (2 μg); lane 8, first fraction of proteins eluted with 250 mM imidazole (5 μg); lane 9, second fraction of proteins eluted with 250 mM imidazole (1 μg); and lane 10, molecular mass markers.

The D238A mutant of the hFADS6 was constructed as described in Materials and Methods. The protein overexpressed in *E. coli* showed similar electrophoretic mobility to that of the WT [21] when purified by Ni-chelating chromatography, i.e., an apparent molecular mass of about 35 kDa. This value was compatible with the theoretical mass derived from the tagged sequence of the expressed polypeptide (38.222 kDa, Figure 3).

**Figure 4:**
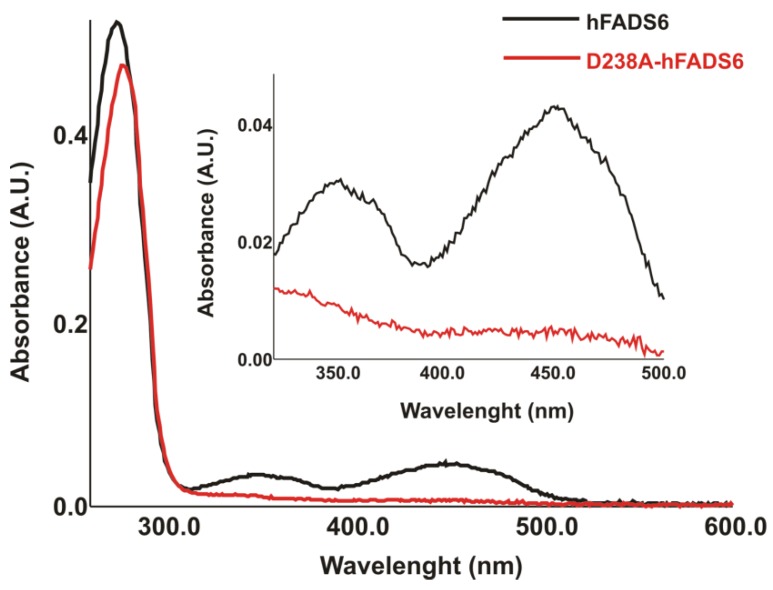
Absorption spectra of hFADS6 and D238A-hFADS6 purified to homogeneity. The spectra of WT hFADS6 (8.6 μM, grey dotted line) and of D238A-hFADS6 (9.7 µM, black line) were recorded in 40 mM HEPES/Na, 5 mM β-mercaptoethanol, pH 7.4. The protein concentration was measured as indicated in [21].

The overexpressed protein was recovered in the soluble fraction of the cell lysate and was used for spectrophotometric analysis and compared with the WT protein. The spectrum of the most abundant purified mutant protein fraction (fraction 8), whose purity is higher than 98%, is shown in Figure 4. An absorbance peak at 274 nm was observed, but surprisingly, no other peaks were present at higher absorption values. As the control, the WT hFADS6 spectrum was shown, presenting two additional minor peaks, at 350 and 450 nm, typical of oxidized FAD (Figure 4). From the absorbance value at 280 and 450 nm a ratio FAD/hFADS6 of 0.43 was calculated, as reported in [21], and thus indicating the presence of some apoprotein. The spectra of fractions 5, 6, 7, and 9 showed the same features as fraction 8. The spectral features of the D238A mutant hFADS6 indicated that this protein does not stabilize the strong binding of flavins.

### 2.3. Kinetics of the D238A hFADS6

**Figure 5:**
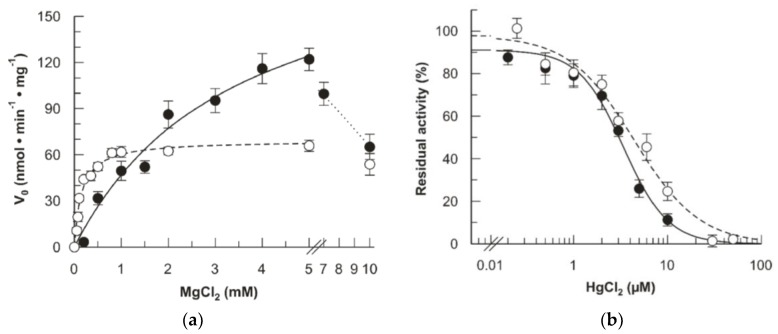
Fluorimetric evidence of FAD synthesis. The FAD synthesis reaction was started by the addition of purified recombinant proteins (hFADS6 open circle or D238A-hFADS6 closed circle) and measured by the initial rate of fluorescence decrease (λ excitation = 450 nm, λ emission = 520 nm). (**a**) Dependence on the MgCl_2_ concentration. FAD synthesis rate, catalyzed by purified hFADS6 (open circle, 10 µg, 0.26 nmoL) or D238A-hFADS6 (closed circle, 2.3 µg, 0.06 nmol), was fluorimetrically measured at 37 °C in 2 mL of 50 mM Tris/HCl pH 7.5, in the presence of 100 µM ATP, 2 µM or 4 µM FMN, respectively, and of the given MgCl_2_ concentrations. (**b**) Inactivation by the mercurial reagent HgCl_2_. FAD synthesis rate, catalyzed by purified hFADS6 (open circle, 10 µg, 0.26 nmoL) or D238A-hFADS6 (closed circle, 3.2 µg, 0.08 nmol), was fluorimetrically measured at 37 °C in 2 mL of 50 mM Tris/HCl pH 7.5, in the presence of 2 µM or 3 µM FMN, respectively, 100 µM ATP, 5 mM MgCl_2_ and of the given HgCl_2_ concentrations. The values of V_0_ are reported as nmol min^−1^ mg^−1^ (**a**) and as percentages of the maximum rate (**b**) arbitrarily set to 100%. Data points are fitted according to the Michaelis–Menten equation (**a**) and according to the IC_50_ equation (**b**) with Grafit 3.0 software.

The mutant protein was characterized in terms of kinetics to uncover possible variations of interactions with the substrates or the effectors and inhibitors. Figure 5 shows the response of the mutant to Mg^2+^, which is a known effector of hFADSs, including the hFADS6 isoform [21,23]. The presence of Mg^2+^ is also essential for activity in the mutant. However, a different behavior was observed. The AC_50_ (half maximum activation constant) of Mg^2+^ is quite higher in the mutant than in the WT. Its value 3.5 ± 0.9 mM is twenty-fold that of the WT. Moreover, a higher value of V_max_ is measured in the case of the mutant. Another distinctive feature is the inhibition observed at higher Mg^2+^ concentrations, which was not present in the WT. The effect of Hg^2+^ was also studied because this heavy metal typically inhibits the WT protein due to interaction with the Cys residues [18,21,23]. Differently from the case of Mg^2+^, the effect of Hg^2+^ on the mutant was very similar to that on the WT enzyme.

**Figure 6:**
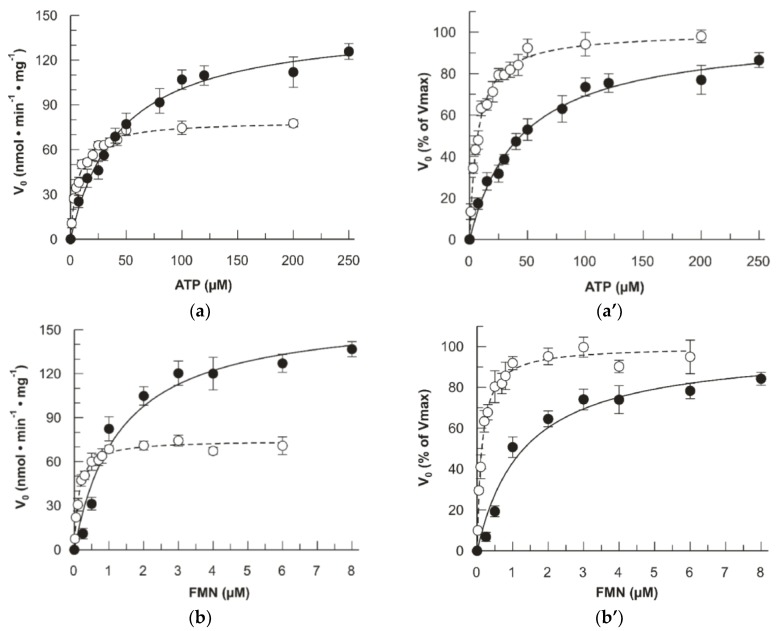
Fluorimetric evidence of FAD synthesis. The FAD synthesis reaction was started by the addition of purified recombinant proteins (hFADS6 open circle or D238A-hFADS6 closed circle) and measured by the initial rate of fluorescence decrease (λ excitation = 450 nm and λ emission = 520 nm). V_0_ was expressed as nmol min^−1^ mg^−1^ (**a**,**b**) and as a percentage of the V_max_ value (**a’**,**b’**) set arbitrarily to 100%. Data points are fitted according to the Michaelis–Menten equation with Grafit 3.0 software. (**a**) ATP concentration dependence. FAD synthesis rate, catalyzed by purified hFADS6 (open circle, 10 µg, 0.26 nmoL) or D238A-hFADS6 (closed circle, 4.2 µg, 0.11 nmoL), was fluorimetrically measured at 37 °C in 2 mL of 50 mM Tris⁄HCl pH 7.5, in the presence of 5 mM MgCl_2_, 2 µM or 3 µM FMN, respectively, and of the given ATP concentrations. (**b**) FMN concentration dependence. FAD synthesis rate catalyzed by purified hFADS6 (open circle, 10 µg, 0.26 nmoL) or D238A-hFADS6 (closed circle, 3.9 µg, 0.10 nmoL) was fluorimetrically measured at 37 °C in 2 mL of 50 mM Tris⁄HCl pH 7.5, in the presence of 5 mM MgCl_2_, 100 µM ATP, and of the given FMN concentrations.

Then, the dependence of the FAD synthesis rate on the concentrations of the main substrates FMN and ATP was studied in the presence of saturating concentration of the counter-substrate, i.e., ATP and FMN, respectively. As shown in Figure 6a, dependences on the ATP concentration for the WT and the mutant were quite different. Both the K_m_ and the V_max_ increased in the mutant. The K_m_ for ATP increased from 6.9 ± 0.5 µM (WT) to 44 ± 4 µM (mutant). The V_max_ increased from 79 ± 1 (WT) to 145 ± 5 nmol min^−1^ mg^−1^ protein. The derived k_cat_ of the mutant was 0.093 ± 0.003 s^−1^ (5.6 ± 0.2 min^−1^) which is about double than that of the WT 0.050 ± 0.001 s^−1^ (3.0 ± 0.1 min^−1^) [21]. To graphically highlight the difference in K_m_, the same data were expressed as percentage of the V_max_ (Figure 6a’). A similar behavior was observed when measuring the dependence of the reaction rate on FMN concentrations. Again, the K_m_ for FMN and the V_max_ increased in the mutant (Figure 6b). The K_m_ for FMN increased from 0.13 ± 0.01 µM (WT) to 1.3 ± 0.3 µM (mutant). The V_max_ increased from 74 ± 1 (WT) to 162 ± 12 nmol min^−1^ mg^−1^ protein (mutant). The derived k_cat_ of the mutant was 0.103 ± 0.007 s^−1^ (6.2 ± 0.5 min^−1^) which is about twice that of the WT 0.047 ± 0.001 s^−1^ (2.9 ± 0.1 min^−1^) [21], very similar to that obtained for the ATP kinetics. In this case, the same data were represented as percentage of the V_max_ (Figure 6b’). Table 1 summarizes the kinetic comparison between WT and D238A hFADS6.

**Figure 7:**
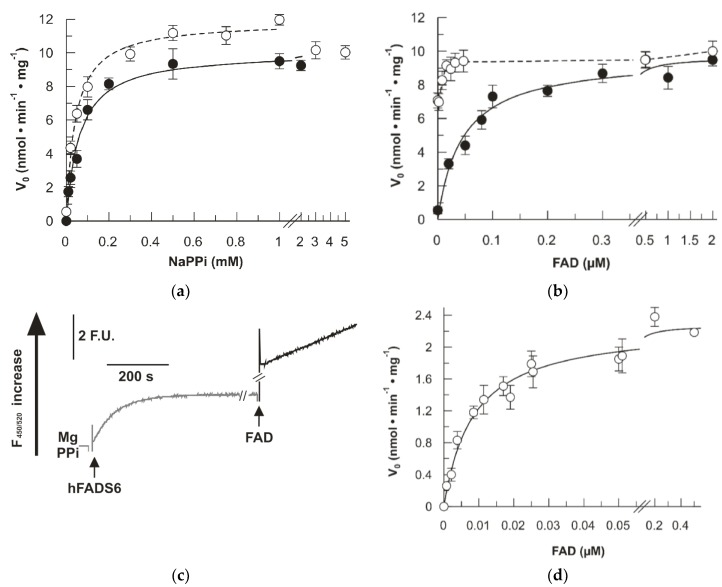
Fluorimetric evidence of FAD cleavage. The FAD cleavage (i.e., pyrophosporolysis) reaction was started by the addition of purified recombinant proteins (hFADS6 open circle, or D238A-hFADS6 closed circle) and measured by the initial rate of fluorescence increase (λ excitation = 450 nm and λ emission = 520 nm). Data points are fitted according to the Michaelis–Menten equation with Grafit 3.0 software. (**a**) NaPPi concentration dependence. FAD cleavage (i.e., pyrophosporolysis) rate, catalyzed by purified hFADS6 (open circle, 10 µg, 0.26 nmol) or 6His-D238A-hFADS6 (closed circle, 10 µg, 0.26 nmol), was measured fluorimetrically at 37 °C in 2 mL of 50 mM Tris ⁄ HCl pH 7.5, in the presence of 5 mM MgCl_2_, 0.5 µM FAD, and of the given NaPPi concentrations. (**b**) FAD concentration dependence. FAD cleavage (i.e., pyrophosporolysis) rate, catalyzed by purified hFADS6 (open circle, 10 µg, 0.26 nmol) or D238A-hFADS6 (closed circle, 10 µg, 0.26 nmoL), was measured fluorimetrically at 37 °C in 2 mL of 50 mM Tris ⁄ HCl pH 7.5, in the presence of 5 mM MgCl_2_, 1 mM NaPPi, and the given FAD concentrations. (**c**) Exogenous FAD cleavage following endogenous FAD removal from hFADS6. The reaction catalyzed by purified hFADS6 (5 µg, 0.13 nmoL) was followed at 37 °C in 2 mL of 50 mM Tris⁄ HCl pH 7.5, in the presence of 5 mM MgCl_2_ and 1 mM NaPPi until the fluorescence reached a constant value corresponding to complete endogenous FAD conversion to FMN (grey line). When indicated exogenous FAD (0.5 µM) was added to calculate the rate of pyrophosporolysis (black line). (**d**) FAD concentration dependence of hFADS6 apo-form. FAD cleavage rate, catalyzed by apo-form of hFADS6 (5 µg, 0.13 nmoL), was measured fluorimetrically at 37 °C in 2 mL of 50 mM Tris ⁄ HCl pH 7.5, in the presence of 5 mM MgCl_2_, 1 mM NaPPi, and the given added FAD concentrations as described in Material and Methods (paragraph 4.6.) and graphically explained in (**c**).

The reverse reaction, i.e., pyrophosphorolysis was also revealed by using recombinant FADS enzymes [21,23]. The kinetics of this reverse reaction was measured for the mutant in comparison with the WT. In the reverse reaction, the K_m_ for NaPPi (Figure 7a) and for FAD (Figure 7b) increased in the mutant, while the V_max_ remained very similar. Even though an increase of K_m_ for FAD (0.045 ± 0.008 µM) is clearly evident in the mutant, the K_m_ value for the WT could not be accurately measured due to both instrumental limitations and to the presence of tightly bound FAD in the active site of the WT protein [21,23]. Therefore, to measure a reliable K_m_ of FAD for the WT, the apo-enzyme was prepared autocatalytically, i.e., by incubating the purified protein at 37 °C for 10 min in the presence of MgCl_2_ and NaPPi and in the absence of externally added FAD, allowing the reverse reaction to occur using the endogenous bound FAD. At this stage, after endogenous FAD conversion to FMN, external FAD was added, and the reverse reaction started (Figure 7c). Under this condition, in which the reaction rate could be measured, being much lower than in the case of the holo-enzyme and, hence, an accurate value of K_m_ could be calculated. As shown in Figure 7d, the K_m_ for FAD of the WT was derived from the curve of Figure 7d. Its value was 0.0079 ± 0.0017 µM. This data correlates well with the data of Figure 7b, confirming that, indeed, the K_m_ of the mutant is much higher.

Since some nucleotides have been proposed to affect the enzyme activity of FADS [20], we have also tested the effect of GTP and NAD^+^ on the mutant activities. The D238A-hFADS6 was inhibited by GTP and by NADH in analogy with the typical behavior of a FADS enzyme (Supplementary Figure S1a,b).

### 2.4. Impact of the D238A Mutation in the Binding Kinetics of Flavinic Substrates

**Figure 8:**
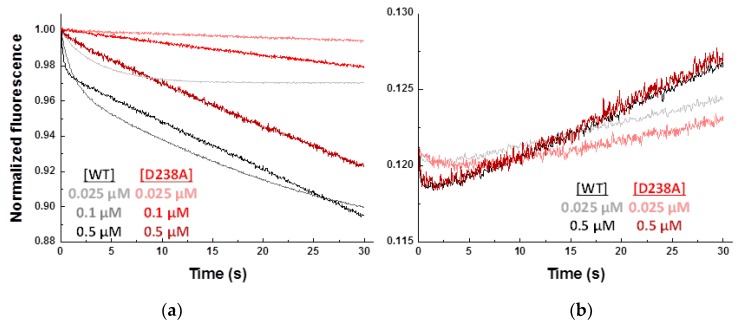
Dissection of the kinetics of the substrates binding process from the catalytic reaction by stopped-flow spectrophotometry. Normalized kinetic traces for the flavin fluorescence evolution upon mixing the protein with substrates for the (**a**) forward (ATP + FMN) and (**b**) reverse (PPi + FAD) reactions. Traces for WT and D238A hFADS are shown in colored grey and red scales as a function of the flavin given concentrations. Normalized signals regarding the fluorescence of the forward reaction at maximum FMN concentration are shown. Kinetic traces were obtained at 25 °C in mixtures containing 100 nM of protein and 250 µM of either ATP (**a**) or PPi (**b**) in 50 mM HEPES/NaOH, 10 mM MgCl_2_, pH 7.0, 5 mM β-mercaptoethanol. All concentrations are final after mixing.

In the attempt to kinetically dissect the binding of substrates from the overall catalytic reaction process we used stopped-flow spectrophotometry to evaluate the forward (ATP + FMN) and reverse (PPi + FAD) reactions in both WT and D238A hFADS6. Binding and catalysis processes might induce changes in the fluorescence of the flavin isoalloxazine; the first due to changes in its electronic environment [25,26], and the second because of the lower FAD fluorescent yield. No relevant changes in flavin fluorescence are detected when mixing WT or D238A hFADS6 with FMN, FAD, or a mixture containing FMN and ADP. These results agree with isothermal titration calorimetry (ITC) lacking to detect direct FMN binding and just envisaging slow direct binding of FAD (Supplementary Figure S2). On the contrary, when introducing the stopped-flow mixtures, in addition to the flavin substrate, the second substrate of the FMN-AT or FADpp activities, ATP or PPi, respectively, noticeably changes in the fluorescence of the flavins are detected (Figure 8).

In mixtures containing substrates of the forward reaction, ATP and FMN, the relevant decrease in the flavin fluorescence is indicative of internalization of the isoalloxazine or FMN transformation into FAD. These observations also agree with ATP binding to the protein prior to FMN in the bi-bi ordered kinetic mechanism reported for hFADSs [17]. Nonetheless, features for this fluorescence decay also differ between WT and D238A hFADSs (Figure 8a). WT hFADS6 shows an initial biexponential fluorescence decay followed by a linear one. The first exponential decay has observed rate constants (k_obs1_) in the low seconds range which depend linearly on the FMN concentration, while its amplitude is concentration independent (Supplementary Figure S3a,b). k_obs2_ for the second exponential decay indicate a slower process nearly FMN concentration independent, with amplitude increasing with FMN concentration (Supplementary Figure S3a,b). Linear dependence of k_obs1_ on substrate concentration indicates this process relates to FMN binding to the protein, and determines the kinetic constants for FMN binding (k_on_ = 3.4 ± 0.1 µM^−1^ s^−1^) and dissociation (k_off_ = 0.32 ± 0.001 s^−1^) for the WT hFADS6:ATP complex, as well as a dissociation constant (K_d_^FMN^ = k_off_/k_on_) for the ternary hFADS6::ATP:FMN complex (Table S1). The derived K_d_^FMN^, 0.094 ± 0.004 µM, agrees well with the above derived K_m_^FMN^ for WT hFADS6. Features of the second exponential process suggest it might represent reorganization of the initial complex to achieve the catalytic organization. Finally, the continuous and linear flavin fluorescence decay established after the exponential processes fits well with the settlement of the catalytic steady-state transformation of FMN into FAD, showing, in addition, rates in the ranges of V_max_ and k_cat_. When similarly evaluating D238A hFADS6, the exponential flavin fluorescence decays related to FMN binding collapse in a single process that becomes hardly detectable, suggesting a deleterious effect of the mutation in the isoalloxazine internalization within the protein. Nonetheless, fluorescence decay related to FMN transformation into FAD follows similar traits to the WT ones (Figure 8a). Kinetic parameters for FMN binding to D238A hFADS6:ATP are as a consequence difficult to unambiguously quantify (Table S1), but data make clear that binding of FMN to the mutant is considerably slower and less efficiently than to WT (Figure S3a). Collectively, these observations further confirm that the D238A mutation has a deleterious effect in the FMN binding process.

When similarly evaluating the reverse reaction, kinetic traces for both WT and the D238A mutant show an initial fluorescence exponential decay of small amplitude that is followed by a linear increase of the signal (Figure 8b). We again deduce the kinetics for binding of FAD from the exponential decay, while rates for the subsequent linear fluorescence increase relate well to steady-state kinetic parameters for the transformation of FAD into FMN. Although the small amplitudes for FAD binding make it difficult to accurately determine *k_obs_* values, both parameters show a dependence on the substrate concentration (Supplementary Figure S3a,b). Errors in estimated kinetic parameters when using these data are high, but obtained values are again indicative of the mutant binding FAD slower and weaker than the WT (Supplementary Table S1 and Figure S3c,d).

## 3. Discussion

The substitution of the D238 residue with A in hFADS6, reproduces the previously described mutation of the homologous residue of the *Cg* FMN-AT that generated an enzyme exhibiting a higher rate of FAD production. The hFADS6 mutant shows similar kinetic changes. The data correlate well with the high conservation of the FAD binding site of eukaryotic FAD synthesizing enzymes along the different species as previously highlighted [16]. This work provides the first evidence that the human FAD synthesizing domain, which corresponds to the hFADS6 enzyme isoform, can be engineered improving its k_cat_ as in the case of the lower eukaryotic organism. Although the k_cat_ value increases, currently, only two-fold, this result can be a promising starting point for further studies based on computational analysis (molecular dynamics) together with additional site-directed mutagenesis to obtain an engineered enzyme with optimized kinetics.

On the basis of changes in both ATP and FMN K_m_s measured here in the forward reaction and of previous observations [17], the binding of the two substrates appears to be synergistic.

Indeed, the extremely low K_m_ for FAD, measured in the course of the reverse reaction, indicates the presence of a very stable bond between FAD. This is in agreement with the finding that size exclusion chromatography is not able to remove FAD from the protein [21,22]. However, the presence of a covalent linkage was previously excluded on the basis of the acidic treatment [23]. Similar strong but non-covalent interactions have been well described for the mitochondrial ADP/ATP binding to carboxyatractilosyde [27]. Indeed, a reliable K_m_ value has only been measured in the present work after using a strategy for removing the bound cofactor (see Figure 7d).

The residue 238 has a major role in this strong interaction. The molecular bases of the improved synthesis rate rely in the correlation between the K_m_ for the FAD and the increase in k_cat_. Indeed, the increased K_m_ derives by a weaker binding of the FMN or FAD to the enzyme active site. This correlates well with the binding data. These experimental evidences confirm the hypothesis that a labile binding allows a faster release of the newly formed cofactor, and hence an increase of the rate of synthesis. The mechanism was firstly hypothesized in the case of the *C. glabrata* enzyme [24]. In this work, in addition to confirming the kinetic data at the basis of the hypothesis, we provide further evidences based on the variations of the binding after FAD conversion into FMN. Moreover, using the stopped-flow procedure that allows dissecting the binding from the overall catalytic process we provide evidences that the increase of K_m_ observed in the mutant can be attributed mostly to a lower binding affinity in the mutant. This could be the basis of a faster release of FAD.

**Figure 9:**
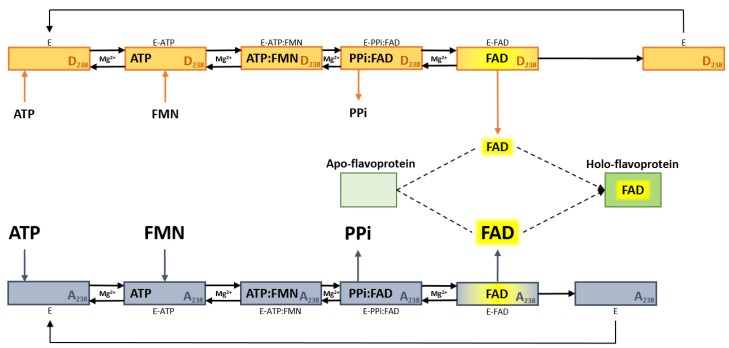
Sketch of the catalytic cycle of FAD synthases. In the figure the schematic representation of FAD synthesis performed by hFADS6 (orange boxes) or hFADS6-D238A (blue boxes), and next cofactor delivery to client apo-flavoprotein (green boxes). Both recombinant proteins show the same catalytic cycle, in addition, several differences are highlighted in the picture: in the mutant, the increased K_m_s for substrates ATP and FMN (in the forward reaction) and PPi and FAD (in the reverse reaction) are highlighted by increasing font size; the increased k_cat_ in the mutant is highlighted by decreasing arrow length in the last step from the enzyme-FAD (E-FAD) complex to free enzyme (E); the increased ability, in the mutant, to deliver the cofactor to client apo-flavoprotein is highlighted by decreasing arrow length from the enzyme-FAD (E-FAD) complex to product FAD. This picture is based on results obtained with the isoform 2 in [17] and with D238A-hFADS6 in this work.

On a structural point of view, the substitution of a hydrophilic residue (Asp) with a hydrophobic one (Ala) weakens the bonds among the FAD molecule and the amino acid residues of the protein active site. The weakening is explained at the molecular level by some structural changes. First, putative hydrogen bonds that fixed FAD to the site are abolished upon substitution of Asp (which can form hydrogen bonds) by Ala (whose side chain is hydrophobic). In addition, the decrease of the side chain size of the 238 residues, resulting from substituting Asp by Ala, favors the release of the cofactor to the external solvent (see Figure 2). This is in agreement with the proposal, which is depicted in the sketch of Figure 9, that the limiting step of the overall catalytic process is the FAD releasing step. This hypothesis has been previously put forward on the bases of steady-state kinetics and cofactor release dynamics in the case of isoform 2 [17,23].

The described changes at the molecular level only affect the active site and its relationships with the cofactor. Indeed, other typical features of the enzyme, such as the inhibition by GTP is similar to the FADS WT enzymes [22,23]. Interestingly, NADH, but not NAD, inhibits the rate of FAD synthesis, thus suggesting an interconnection between the two cofactors and the ability to discriminate between the redox state of nicotinamide pool. The discrimination may be related to the positive charge of the nicotinamide ring. The same features were demonstrated with isoform 2; when measuring FAD hydrolase activity NADH is also substrate of FADHy domain [20].

Again, the response to the SH binding reagent HgCl_2_, is not affected by the mutation, correlating with the previous finding that the SH sensitive residue(s) are not in close proximity to the active site [18]. This phenomenon of k_cat_ increases also introduces important perspectives in human health. The hFADS6 has been previously described as an “emergency protein” on the basis of its expression in patients with FADS gene defects [7] and of its functional and biochemical characterization [21]. In these patients, a decrease of FAD synthesis is the cause of the pathological alterations. Thus, the knowledge of a strategy to increase the reaction rate, i.e., the capacity of synthesizing FAD, of the emergency protein hFADS6, could open the possibility to ameliorate therapeutic interventions. As an example, targeting the D238 residue by specific chemical reagents (drugs) that reduce the hydrophilic property of this residue could partly mimic the effect of the mutation. Chemical screening and further mutations are under investigation to find conditions for further increasing the FAD synthesis rate of hFADS6.

## 4. Materials and Methods

### 4.1. Materials

All chemicals were from Sigma-Aldrich (St. Louis, MO, USA), unless otherwise specified. The *Escherichia coli* Rosetta(DE3) strain was purchased from Novagen (Madison, WI, USA). Restriction endonucleases and other cloning reagents were purchased from Fermentas (Glen Burnie, MD, USA). Chelating Sepharose Fast Flow was from Amersham Biosciences (Arlington Heights, IL, USA), and the Isolate II PCR and Gel Kit (Bioline, London, UK). The dye reagent for protein assay was from Bio-Rad (Hemel Hempstead, Herts, UK).

### 4.2. Site-Directed Mutagenesis of the hFADS6

The hFADS6-D238A mutant was obtained by PCR overlap extension method [18] using as forward and reverse mutagenic primers 5′-TTCAGCCCCACTGCTCCAGGCTGGCCCGCATTCAT-3′ and 5′-ATGAATGCGGGCCAGCCTGGAGCAGTGGGGCTGAA-3′, respectively. The external forward and reverse wild type primers allowed the directional cloning of the desired hFADS6-D238A cDNA between *Eco*RI (at 5′ end) and *Xho*I (at 3′ end) restriction sites of the pH6EX3 expression vector. The resulting recombinant protein carried the extra N-terminal sequence MSPIHHHHHHLVPRGSEASNS.

### 4.3. Expression of the WT hFADS6 and hFADS6-D238A Proteins in E. coli

Rosetta(DE3) strain was transformed with either pH6EX3-hFADS6 or pH6EX3-hFADS6-D238A plasmids by calcium chloride treatment. Selection of transformed colonies was performed on LB-agar plates containing 100 µg/mL ampicillin and 34 µg/mL chloramphenicol. *E. coli* Rosetta(DE3) cells carrying the recombinant plasmids were inoculated in 10 mL of LB medium (1% tryptone, 0.5% yeast extract, 0.5% NaCl, pH 7.0) supplemented with 100 µg/mL ampicillin and 34 µg/mL chloramphenicol, and cultured overnight at 37 °C with rotary shaking (~180 rpm). The day after, a 5 mL aliquot of the cell culture was transferred to 0.5 L of fresh LB medium supplemented with antibiotics and grown at 37 °C to A600 equal to 0.8 to 1. Then, 0.5 mM IPTG was added to induce the expression of the recombinant WT and mutant proteins. Growth was continued overnight at 20 °C, bacteria were harvested by centrifugation at 3000× *g* for 10 min at 4 °C and the pellets stored at −20 °C. The bacterial pellet (about 4 g wet weight) was thawed on ice and resuspended in 30 mL start buffer (500 mM NaCl, 40 mM HEPES/Na, pH 7.4) supplemented with protease inhibitor cocktail (P8849, Sigma-Aldrich, Merck, Milan, Italy 1 mL/20 g of cells wet weight) and 0.5 mM phenylmethyl sulfonyl fluoride (PMSF, P7626, Sigma-Aldrich, Merck, Milan, Italy). Cells were disrupted by mild sonication (one cycle of 10 min and one cycle of 5 min with 1 s Pulse ON and 1 s Pulse OFF, at 40 W) on ice bath using a VCX-130 Sonifier (Sonics). The soluble and the insoluble cell fractions were separated by centrifugation of the cell lysate at 20,000× *g* for 30 min at 4 °C. The supernatant, containing the soluble overexpressed 6His-hFADS6 or 6His-D238A-hFADS6 proteins, was used for SDS-PAGE analysis, FADS activity assay and further protein purification (see below).

### 4.4. Purification of Recombinant hFADS6-D238A

A 40 mL aliquot of the soluble cell fraction, obtained from *E. coli* Rosetta(DE3) strain transformed with the pH6EX3-hFADS6-D238A plasmid, was applied onto a Chelating Sepharose Fast Flow column (3.5 mL packed resin), previously charged with 200 mM NiSO_4_ according to the producer’s protocol, and equilibrated with the start buffer. The column was first washed with 35 mL start buffer, then, developed with a step gradient of 50 mM, 100 mM, 250 mM, and 500 mM imidazole in the same buffer. At each step of the purification procedure, the FADS activity was measured (see below) and the purity of the proteins was checked by SDS-PAGE. Prior to storing or further processing, fractions containing the purified recombinant protein were desalted by gel filtration on a PD10 column in 40 mM HEPES/Na, 5 mM β-mercaptoethanol, pH 7.4. These protein samples were stable for at least 30 days at 4 °C.

Protein concentration and FAD/protein monomer ratio measurements were carried out as previously described [21].

### 4.5. 3D Modeling and Docking of the WT hFADS6 and hFADS6-D238A Proteins

The 3D model of the hFADS6 protein was obtained by ab initio using the server Robetta (available at http://robetta.bakerlab.org/) as previously described [21]. FAD was docked in the same position as in the 3G6K structure by aligning the two structures with the MatchMaker tool of the Chimera software using Needleman-Wunsch algorithm and BLOSUM-62 matrix [28]. D238A mutation in hFADS6 structure was introduced using a rotamer library according to Shapovalov and Dunbrack [29].

### 4.6. Measurements of Enzyme Catalyzed Rates for FAD Synthesis and FAD Pyrophosphorolysis

The rate of FAD synthesis and FAD cleavage were measured in continuo as in [23], by exploiting the different fluorescence properties of FAD with respect to FMN. Fluorescence time courses (λ excitation at 450 nm and λ emission at 520 nm) were followed at 37 °C in a FP-8300 Jasco spectrofluorometer. In each experiment, FMN and FAD fluorescence were calibrated by using standard solutions whose concentrations (used in µM range) were calculated by using ε_450_ of 12.2 mM^−1^ cm^−1^ for FMN and 11.3 mM^−1^ cm^−1^ for FAD. Under the experimental condition used here, the specific relative FAD fluorescence coefficient (ϕ_FAD_) proved to be about ten times lower than those of FMN (ϕ_FMN_) [30,31].

For FAD synthesis rate measurements, purified protein fractions (2 to 10 µg, 0.06 to 0.26 nmoL protein as monomer, unless otherwise indicated) were incubated in 50 mM Tris⁄HCl, pH 7.5, containing 5 mM MgCl_2_, 3 µM FMN, 100 µM ATP, and additional reagents as appropriate. The rate of FAD synthesis, expressed as nmoL FAD min^−1^ mg protein^−1^, was calculated from the rate of fluorescence decrease, measured as the tangent to the initial part of the experimental curve by applying the following equation:

V_0_ = [(ΔF_450/520_/Δϕ_450/520_) × Vf]/(t × m) (1)

where ΔF is expressed in fluorescence arbitrary units, Δϕ = ϕ_FMN_ − ϕ_FAD_ is expressed as µM^−1^, Vf is expressed in mL, t is time expressed in min, and m is the mass of protein in mg.

The rate of FAD pyrophosphorolysis catalyzed by 6His-D238A-hFADS6 (5 to 10 µg, 0.13 to 0.26 nmoL protein as monomer) was measured in 50 mM Tris⁄HCl, pH 7.5, containing 5 mM MgCl_2_ in the presence of 5 mM MgCl_2_, 1 mM NaPPi (sodium pyrophosphate), and 0.5 µM FAD, unless otherwise indicated. The rate of FAD cleavage was expressed as nmol FAD min^−1^ mg protein-1, and was calculated from the rate of fluorescence increase, measured as the tangent to the initial part of the experimental curve, as previously described.

In the case of using WT hFADS6, which is a FAD binding protein, the measurement of Km for FAD might not be accurate. To overcome this limitation, the apo-form of the protein was obtained by a rapid procedure in the cuvette simply by allowing autocatalytic endogenous bound FAD conversion to FMN by adding only 1 mM NaPPi. The reaction was followed for about 10 min until endogenous bound FAD is completely converted to FMN, i.e., the fluorescence does not change. At this stage, external FAD was added at different concentrations and the dependence of the reaction rate was studied.

### 4.7. Kinetics for the Binding of Flavinic Ligands to WT hFADS6 and hFADS6-D238A Proteins

Pre-steady-state kinetic experiments were performed using stopped-flow spectroscopy on an *Applied Photophysics* SX17.MV spectrophotometer using the Pro-Data SX software (Applied Photophysics Ltd. Leatherhead, Surrey, UK). The fluorescence of flavins was measured in a continuous assay with an excitation wavelength of 445 nm, while the emission was recovered using a >530 nm cut-off filter and the voltage was set to 350 V. Then, 100 nM hFADS6 samples were mixed with samples that contained increasing concentrations of FMN or FAD (which varied in the range 0.025–1 μM), in the absence and in the presence of saturating concentrations of their respective co-substrates ATP or PPi (250 µM). All the indicated concentrations are final ones in the stopped-flow observation cell. Measurements were carried out at 25 °C in 50 mM HEPES/NaOH, 10 mM MgCl_2_, pH 7.0, 5 mM β-mercaptoethanol. At least three reproducible kinetic traces of changes in fluorescence were recorded for each condition and fitted to exponential equations (usually one or two exponentials were used)

(2) $$y=\sum A_{i}e^{-k_{\mathrm{obs},i}t}$$

where A_i_ and k_obs,i_ represent the amplitude and the observed rate constant, respectively, for each of the processes (i) that contribute to the overall time-dependent fluorescence change for each experimental condition. Processes whose k_obs_ showed a linear dependence on flavin concentration were fit to a one-step model that accounts for the kinetic equilibrium of the formation and dissociation of the protein-flavin complex, whose kinetics can be represented by

(3) $$k_{\mathrm{obs}}=k_{\mathrm{on}}\left[\mathrm{FLV}\right]+k_{\mathrm{off}}$$

where k_on_ and k_off_ are the kinetic constants for complex formation and dissociation, respectively.

### 4.8. Isothermal Titration Calorimetry (ITC)

Measurements were carried out using an Auto-ITC200 microcalorimeter (MicroCal LLC, Northampton, MA, USA) thermostated at 25 °C. Ligand (100 µM FMN or FAD) and proteins (~15 µM) were dissolved in 50 mM HEPES/NaOH, 10 mM MgCl_2_, pH 7.0, 2 mM β-mercaptoethanol, and degassed prior to titration. Up to 19 injections of 2 µL of ligand were added to the sample cell (~0.2 mL) containing the enzyme and then mixed via the rotating (1000 rpm) stirrer syringe. Since either no binding or slow binding was detected, data fitting to obtain thermodynamic binding parameters was not reliable.

### 4.9. Electrophoretic Analysis

Proteins were separated by SDS-PAGE on 12% total polyacrylamide gels, according to Laemmli [32]. Quantitative evaluation of Coomassie blue-stained protein bands was carried out using the Chemidoc imaging system and the Quantity One software (Bio-Rad), as described previously [33].

## Figures and Tables

**Table 1:** Comparison between K_m_s for substrates of hFADS6 and D238A-hFADS6. * The value was obtained using the autocatalytically formation of apo-eznyme as described in Materials and Methods and graphically explained in Figure 7c. Km values of the mutant are in bold

	6His-hFADS6	6His-D238A-hFADS6
	**Forward reaction**	
K_m_ FMN (µM)	0.13 ± 0.01	**1.3 ± 0.3**
K_m_ ATP (µM)	6.9 ± 0.5	**44 ± 4**
Ac_50_ Mg^2+^ (mM)	0.15 ± 0.02	**3.5 ± 0.9**
	**Reverse reaction**	
K_m_ PPi (mM)	0.042 ± 0.006	**0.060 ± 0.008**
K_m_ FAD (µM)	0.0079 ± 0.0017 *	**0.045 ± 0.008**

## Supplementary Materials

Supplementary materials can be found at https://www.mdpi.com/1422-0067/20/24/6203/s1.

## Abbreviations

FADS	FAD synthase
FMNAT	FMN adenylyl transferase
hFADS6	human FAD synthase isoform 6
Rf	riboflavin
FMN	flavin mono nucleotide
FAD	flavin adenine dinucleotide
PAPS	phosphoadenosine 5-phosphosulfate
FADSy	FAD synthase domain
MPTb	molybdopterin binding
FADHy	FAD hydrolase domain
BVVLS	Brown–Vialetto-van Laere syndrome
RR-MADD PMSF	riboflavin responsive multiple acyl-CoA dehydrogenase deficiency phenylmethyl sulfonyl fluoride
