# Supplementary material for: Mutation of Aspartate 238 in FAD Synthase Isoform 6 Increases the Specific Activity by Weakening the FAD Binding
Source: Int J Mol Sci. 2019 Dec 9;20(24):6203. doi: 10.3390/ijms20246203 (PMC6941110; doi:10.3390/ijms20246203)
Supplement: Supplementary file 1 [file ijms-20-06203-s001.pdf]

International Journal of *Molecular Sciences*

MDPI

Article

Mutation of Aspartate 238 in FAD Synthase Isoform 6 Increases the Specific Activity by Weakening the FAD Binding

Piero Leone [1,2], Michele Galluccio [2], Stefano Quarta [1], Ernesto Anoz-Carbonell [3], Milagros Medina [3], Cesare Indiveri [2] and Maria Barile [1,*]

1 Department of Biosciences, Biotechnology and Biopharmaceutics, University of Bari, via Orabona 4 –, 70126 Bari, Italy; pieroleone87@gmail.com (P.L.); quartastefano93@gmail.com (S.Q.)
2 Department of Biology, Ecology and Earth Sciences (DiBEST), Unit of Biochemistry and Molecular Biotechnology, University of Calabria, via P. Bucci 4c, 87036 Arcavacata di Rende, Italy; michele.galluccio@unical.it (M.G.); cesare.indiveri@unical.it (C.I.)
3 Departamento de Bioquímica y Biología Molecular y Celular, Facultad de Ciencias, Instituto de Biocomputación y Física de Sistemas Complejos (GBsC-CSIC and BIFI-IQFR Joint Units), Universidad de Zaragoza, Zaragoza 50009, Spain; eanoz@unizar.es (E.A.-C.); mmedina@unizar.es (M.M.)
* Correspondence: maria.barile@uniba.it

Supplementary Materials: Supplementary materials can be found at www.mdpi.com/xxx/s1.

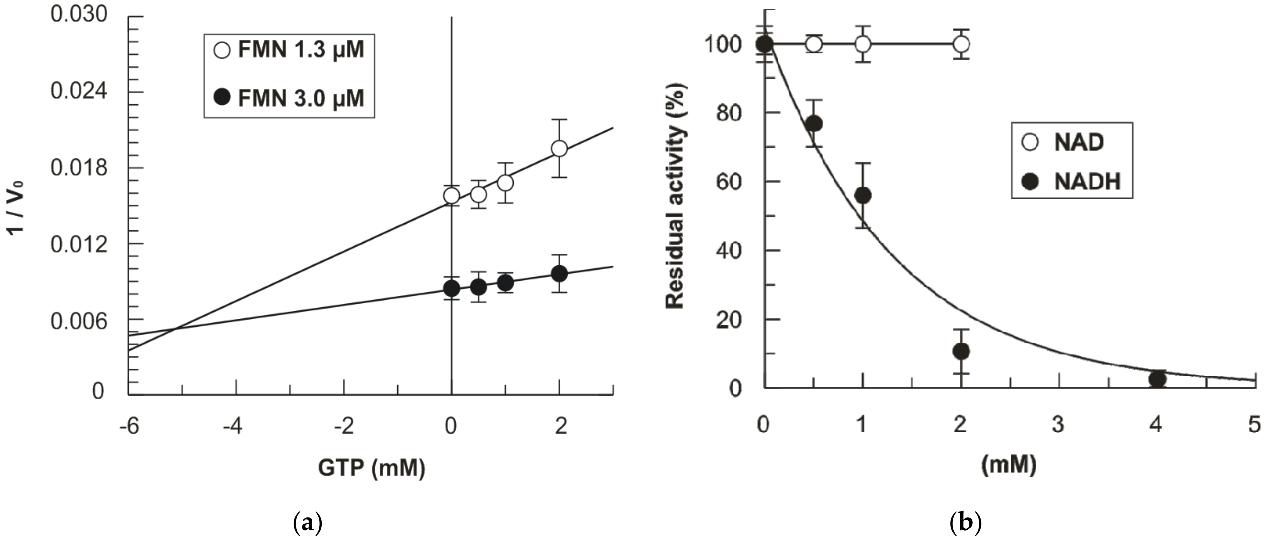

Supplementary Figure 1. (a) GTP inhibition on FAD synthesis catalyzed by D238A-hFADS6. The FAD synthesis reaction was started by the addition of purified recombinant protein D238A-hFADS6 and measured by the initial rate of fluorescence decrease (λ excitation = 450 nm, λ emission = 520 nm). FAD synthesis rate, catalyzed by purified D238A-hFADS6 (2.9 μg, 0.08 nmol), was fluorimetrically measured at 37 °C in 2 ml of 50 mM Tris/HCl pH 7.5, in the presence of 100 μM ATP, 5 mM $MgCl_2$, 3 μM (closed circle) or 1.3 μM (open circle) FMN, and of the given GTP concentrations. Data points are fitted according to the linear equation with Grafit 3.0 software. **(b)** NAD^+ and NADH inhibition on FAD synthesis. The FAD synthesis reaction was started by the addition of purified recombinant protein 6His-D238A-hFADS6 and measured by the initial rate of fluorescence decrease (λ excitation = 450 nm, λ emission = 520 nm). FAD synthesis rate, catalyzed by purified D238A-hFADS6 (8 μg, 0.21 nmol), was fluorimetrically measured at 37°C in 2 ml of 50 mM Tris/HCl pH 7.5, in the presence of 100 μM ATP, 5 mM $MgCl_2$, 1.3 μM FMN, and of the given NAD^+ (open circle) and NADH (closed circle) concentrations. Data points are fitted according to the exponential or linear equations for NADH and NAD^+ respectively with Grafit 3.0 software.

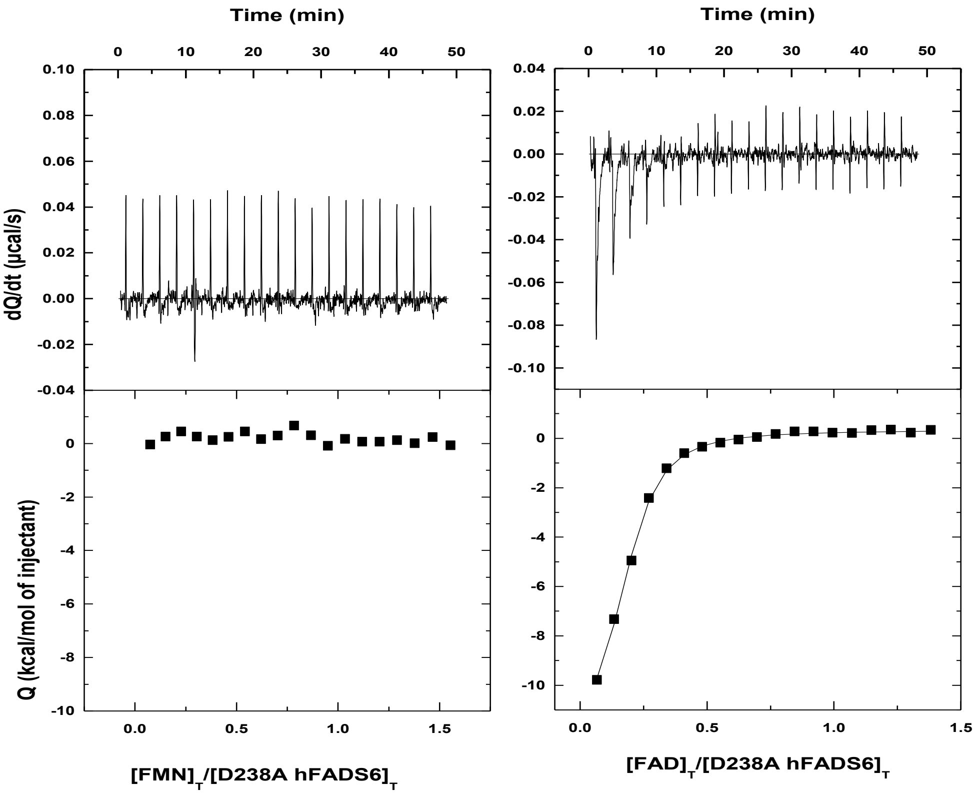

(**a**) (**b**)

Supplementary Figure 2. Isothermal titration calorimetric analysis of the binding of flavinic substrates to D238A hFADS6 in the absence of the second substrate. Thermogram (upper panels) and binding isotherms with integrated heat (lower panels) for the titration with (**a**) FMN and (**b**) FAD. No interaction heat is detected (a) for the titration of FMN, suggesting under the assayed conditions binding is not produced. Thermograms for the titration with FAD titration envisage binding to less than 20% of the protein molecules (suggesting a slow-binding process) under the assayed conditions, as well as K_d^{FAD} below 1 µM. Titrations were performed at 25 °C in 50 mM Hepes/NaOH, 10 mM $MgCl_2$, pH 7.0, 5 mM β-mercaptoethanol. The low stability of WT hFADS6 along the ITC assay prevented production of the corresponding thermograms.

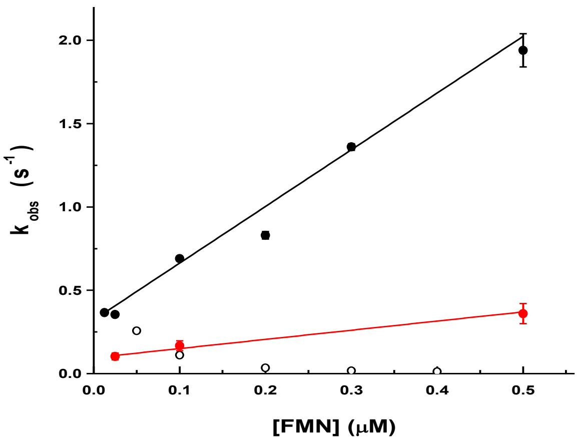

(**a**)

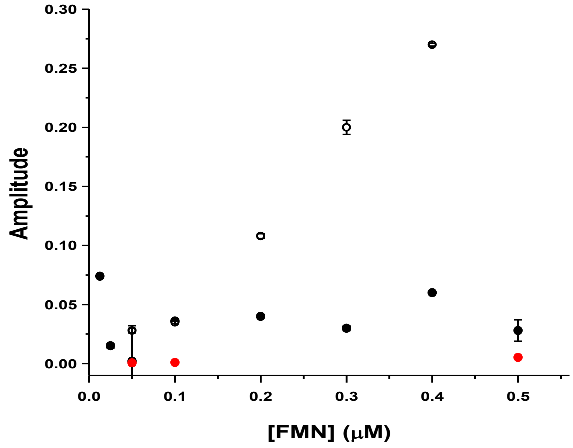

(**b**)

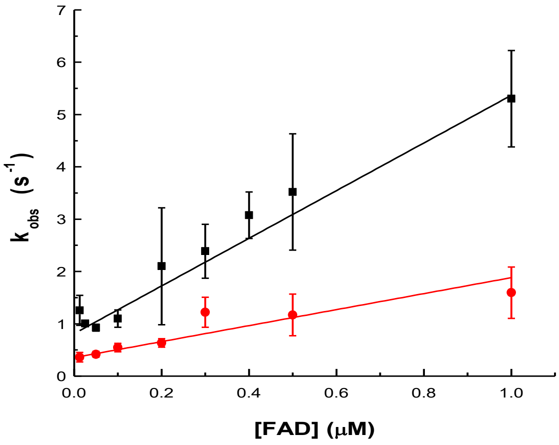

(**c**)

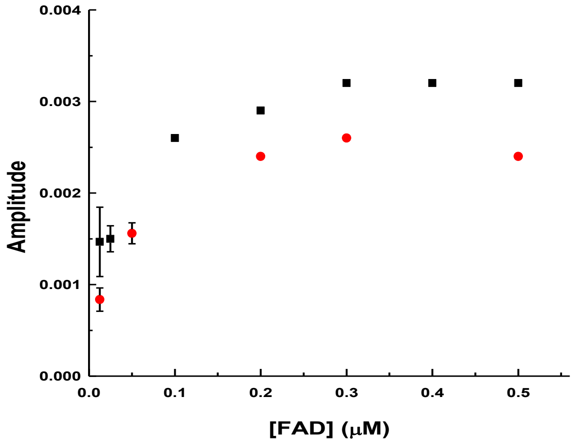

(**d**)

Supplementary Figure 3. Dependence of flavin binding pre-steady-state kinetic parameters on the concentration of the flavin substrates. Evolution of binding parameters for the FAD biosynthesis: (**a**) k_{obs1} (closed circles) and k_{obs2} (open circles) and (**b**) their corresponding amplitudes, Amp1 (closed circles) and Amp2 (open circles), obtained when mixing WT (black) and D238A (red) hFADS6s with saturating ATP and different FMN concentrations. Evolution of binding parameters for the FAD pyrophosphorolysis: (**c**) **k_{obs} (closed circles)** and (**d**) **Amp (closed circles)** obtained when mixing WT (black) and D238A (red) hFADS6s with saturating PPi and different FAD concentrations. Data were obtained at 25 °C in mixtures containing 100 nM of protein and 250 μM of either ATP or PPi (respectively for FAD synthesis and FAD pyrophosphorolysis) in 50 mM Hepes/NaOH, 10 mM $MgCl_2$, pH 7.0, 5 mM β-mercaptoethanol.

Supplementary Table 1. Kinetic parameters derived from stopped-flow data for the binding of FMN and FAD to WT and D238A hFADS6 in the presence of the second substrate (ATP or PPi, respectively). k_{on} and k_{off} the kinetic constants for complex formation and dissociation were obtained by fitting lineal data corresponding to k_{obs1} dependences on flavin concentration presented in Supplementary Figures 3a and 3c (n = 3, mean ± SD). Dissociation constants determined as k_{off}/k_{on}. Data obtained at 25 °C at 25 °C in 50 mM Hepes/NaOH, 10 mM $MgCl_2$, pH 7.0, 5 mM β-mercaptoethanol.

**FMN-ATP mixtures (FMN binding)**			
**hFADS6**	k_{on} ($\mu M^{-1}.s^{-1}$)	k_{off} (s^{-1})	K_d^{FMN} (μM)
**WT**	3.4 ± 0.1	0.32 ± 0.01	0.094 ± 0.004
**D238A**	0.55 [a]	0.09 [a]	>0.18 [a]
**FAD-NaPP$_i$ mixtures (FAD binding)**			
	k_{on} ($\mu M^{-1}.s^{-1}$)	k_{off} (s^{-1})	K_d^{FAD} (μM)
**WT**	4.5 ± 0.6	0.81 ± 0.05	0.18 ± 0.04
**D238A**	1.5 [a]	0.35 [a]	> 0.3 [a]

[a] Error in the determination of parameters for the mutant is high, at least ± 30%, due to small amplitudes in the exponential decays and/or low data reproducibility.
